# Mortality risk after clinical management of recurrent and metastatic adenoid cystic carcinoma

**DOI:** 10.1186/s40463-018-0273-z

**Published:** 2018-04-25

**Authors:** Melody J. Xu, Tara J. Wu, Annemieke van Zante, Ivan H. El-Sayed, Alain P. Algazi, William R. Ryan, Patrick K. Ha, Sue S. Yom

**Affiliations:** 1 Department of Radiation Oncology, University of California San Francisco, San Francisco, CA USA; 2 Department of Head and Neck Surgery, University of California Los Angeles, Los Angeles, CA USA; 3 Department of Pathology, University of California San Francisco, San Francisco, CA USA; 4 Division of Head and Neck Oncologic Surgery, Department of Otolaryngology-Head and Neck Surgery, University of California San Francisco, San Francisco, CA USA; 5 Department of Medicine, University of California San Francisco, San Francisco, CA USA

**Keywords:** Adenoid cystic carcinoma, Recurrence, Skull base, Lung metastases, Survival

## Abstract

**Background:**

Management of locoregional recurrence (LRR) and distant metastasis (DM) in adenoid cystic carcinoma (ACC) is guided by limited data. We investigated mortality risks in patients diagnosed and treated for recurrent ACC.

**Methods:**

A retrospective review of ACC patients treated from 1989 to 2016 identified 36 patients with LRR or DM. High-risk disease was defined as skull base involvement (for LRR) or International Registry of Lung Metastases Group III/IV or extrapulmonary site of metastasis (for DM). Kaplan-Meier method, log-rank tests, and Cox proportional hazards were used for time-to-event analysis.

**Results:**

Among 20 LRR and 16 DM patients, the median times to recurrence were 51 and 50 months, respectively. The median follow-up post-recurrence was 37.5 months (interquartile range (IQR)16.5–56.5). Post-recurrence 3-year overall survival (OS) was 78.5%, 73.3% for LRR and 85.1% for DM (*p* = 0.62). High-risk recurrences were associated with worse 3-year OS (68.8% for high-risk and 92.3% for low-risk, χ2 = 10.4, *p* = 0.001).

Among LRR patients, 90% had surgery as part of their treatment. Multimodality therapy, age, and histopathologic features (size, margins, solid histology, lymphovascular or perineural invasion) were not associated with PFS or OS. High-risk LRR was the only variable associated with OS (χ2 = 5.9, *p* = 0.01).

Among DM patients, six were initially managed with observation and ten received surgery, RT, or systemic therapy. Upfront therapy was not associated with improved PFS or OS. High-risk DM was the only variable associated with OS (χ2 = 4.7, *p* = 0.03).

**Conclusions:**

High-risk LRR and DM were associated with decreased 3-year OS. More effective therapies are needed for high-risk ACC recurrences.

## Background

Adenoid cystic carcinoma (ACC) is an uncommon secretory gland malignancy arising from a variety of head and neck sites, including major and minor salivary glands, palate, maxilla, and trachea [1, 2]. Localized presentations of ACC are managed with surgery with or without postoperative radiation therapy (PORT) to improve local control [1–5].

The locoregional recurrence (LRR) rate is approximately 40% at 5 years, with T4 stage, nodal involvement, solid histology, perineural invasion (PNI) and positive surgical margins considered to be negative prognostic factors [6–10]. Lymph node metastases are uncommon [7, 11]. Distant metastases (DM) eventually occur in as many as 40% of patients, with the lung being the most common site of metastasis [1, 2, 8, 12]. While DM of ACC are traditionally thought to have indolent growth, reports suggest that solid histology, size greater than 3 cm, positive surgical margins, and presence of nodal involvement are associated with a more aggressive disease course [2, 13].

Limited data is available to guide management of LRR and DM. Metastasectomy of pulmonary metastases has been proposed, with promising results in patients with good performance status, young age, and long disease-free interval (DFI) prior to DM [14–18]. Systemic therapy outcomes have been reported in less than 500 patients over 20 years of publications but none have become a dominant standard of care; investigation of more effective therapeutics is needed [19].

To our knowledge, there are no reports focused on analyzing outcomes from local therapies such as surgery and/or radiation for recurrent and metastatic ACC patients. We therefore conducted a retrospective review to describe the LRR and DM management and outcomes of recurrent ACC treated at our institution.

## Methods

### Patient selection

We identified 85 patients with pathology-confirmed diagnosis of ACC treated at our center from 1989 to 2016 who had undergone surgical excision with or without PORT for initial treatment, and who had greater than six months of follow-up after initial treatment. Within this cohort of patients, we identified 36 patients (42.4%) who experienced LRR or DM.

### Definition of variables

LRR was defined as recurrence at the initial site, immediately adjacent area, or regional draining nodal basin. DM was defined by radiographic findings consistent with metastatic disease and was confirmed with biopsy whenever possible. One patient had an orbital LRR and small lung DM diagnosed simultaneously and was treated aggressively at the orbit; this patient was categorized as having LRR as the primary first event after definitive treatment. Pathology reports were used to identify histologic subtype (e.g. tubular, cribriform, solid, etc.), margin status, PNI, and lymphovascular space invasion (LVSI).

High-risk LRR was defined as being at the skull base. High-risk DM was classified as International Registry of Lung Metastases (IRLM) Group III or IV (resectable but with DFI < 36 months and multiple metastases, or unresectable) or extrapulmonary site of metastasis. Older age was defined as age greater than the sample median of 63 years.

For the purposes of this report, follow-up time, progression-free survival (PFS), and overall survival (OS) were calculated from time of recurrence diagnosis. PFS was defined as progression of the initially recurrent tumor or development of new LRR or DM after treatment. PFS was censored at date of death or last follow-up and OS was censored at date of last follow-up in clinic or last known date of contact.

### Statistical analysis

STATA (13th Edition, StataCorp, College Station, TX) was used for statistical analyses. Wilcoxon rank-sum tests were used for comparison of continuous variable distribution between LRR and DM recurrence types and Fisher’s exact tests were used for comparison of categorical variable distribution between LRR and DM recurrence types. Kaplan-Meier method, log-rank tests, and Cox proportional hazards models were used for time-to-event analysis.

## Results

### Recurrence characteristics

Of the 36 identified patients with ACC recurrence, 20 (55.6%) had LRR and 16 (44.4%) had DM. Table 1 shows patient characteristics of this cohort, and Table 2 describes the treatment characteristics and outcome among patients who experienced LRR and DM. The median age at diagnosis for LRR or DM was 63.5 years (range, 30–85). Thirty-four patients (94%) had pathologic confirmation of LRR or DM. The other two patients had evident radiographic disease progression in the lung and cavernous sinus. On histopathologic review of recurrences, PNI was present in 11 recurrences (30.6%) and LVSI was present in four recurrences (11.1%). Histologic subtype was identified in 16 recurrences, with six of them (37.5%) having some solid component in the recurrent specimen.

**Table 1:** Characteristics of patients experiencing locoregional recurrence (LRR) or distant metastasis (DM) as first adenoid cystic carcinoma recurrence

	Overall	LRR	DM	*p* value
Characteristic	36	20	16	
Ethnicity				
White (incl. Hispanic)	25 (69.4%)	10 (50.0%)	15 (93.8%)	0.02
Asian/Pacific-Islander	8 (22.2%)	7 (35.0%)	1 (6.3%)	
African-American	1 (2.8%)	1 (5.0%)	0	
Other	2 (5.6%)	2 (10.0%)	0	
Female	24 (66.7%)	13 (65.0%)	11 (68.8%)	0.55
Male	12 (33.3%)	7 (35.0%)	5 (31.3%)	
Initial Stage				0.38
I-II	5 (22.7%)	1 (12.5%)	4 (28.6%)	
III-IV	15 (77.3%)	7 (87.5%)	10 (71.4%)	
Unknown	14	12	2	
Initial Treatment				
Surgery	7 (19.4%)	6 (30.0%)	1 (6.3%)	0.08
Surgery + PORT	29 (80.6%)	14 (70.0%)	15 (93.8%)	
Age at recurrence, Median (IQR)	64 (44–74)	66 (47–76)	60 (42–70)	0.48
Months to recurrence after initial treatment, Median (IQR)	50 (16–88)	51 (15–108)	50 (18–77)	0.58
Low-risk	14 (38.9%)	9 (45.0%)	5 (31.3%)	0.31
High-risk	22 (61.1%)	11 (55.0%)	11 (68.8%)	

*IQR* interquartile range, *RT* radiation therapy, *PORT* post-operative radiation therapy

**Table 2:** Treatment characteristics and outcomes of patients experiencing locoregional recurrence (LRR) or distant metastasis (DM) as first recurrence of adenoid cystic carcinoma

	LRR (*n* = 20)	*p* value	DM (*n* = 16)	*p* value
Treatment				
Surgery	10		5	
Surgery + PORT	8		0	
RT alone	1		4	
CF-RT	1		0	
SBRT	0		2	
Palliative RT	0		2	
Systemic therapy alone	1		1	
Observation	0		6	
3-year PFS	82.1%		61.2%	
3-year PFS				
Low-risk	100%	0.15	80.0%	0.48
High-risk	64.3%		44.4%	
3-year OS	73.3%		85.1%	
3-year OS				
Low-risk	87.5%	0.01	100%	0.03
High-risk	62.3%		76.2%	

*CF-RT* conventionally fractionated radiation therapy, *RT* radiation therapy, *PFS* progression-free survival, *PORT* post-operative radiation therapy, *OS* overall survival

### Overall outcomes

After recurrence (LRR or DM) diagnosis, the median follow-up time was 37.5 months (interquartile range (IQR), 16.5–56.5). The overall 3-year PFS after diagnosis of recurrence was 71.7%; it was 82.1% for patients with LRR and 61.2% for patients with DM (χ2 = 2.36, *p* = 0.12). Overall 3-year OS was 78.5%; it was 73.3% for patients with LRR and 85.1% for patients with DM (χ2 = 0.24, *p* = 0.62).

**Fig. 1:**
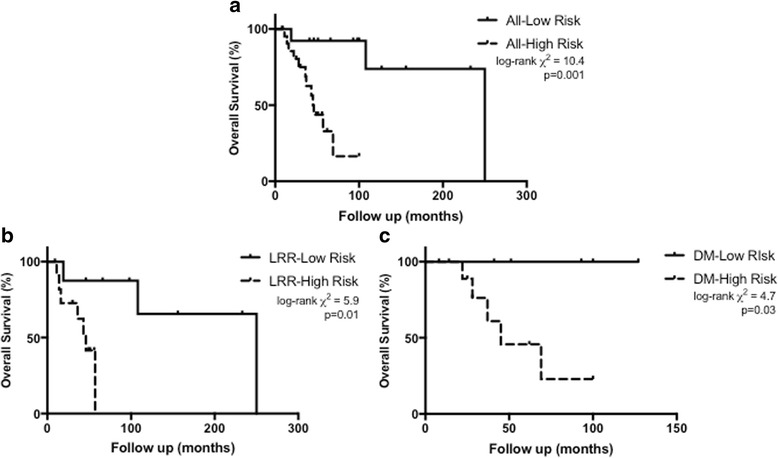
Kaplan-Meier estimated overall survival, comparing outcomes of recurrent adenoid cystic carcinoma among (**a**) all patients with low-risk and high-risk recurrence, (**b**) patients with low-risk and high-risk locoregional recurrence, and (**c**) patients with low-risk and high-risk distant metastasis

High-risk recurrence (skull base LRR, IRLM Group III/IV lung DM, or extrapulmonary DM) was associated with worse OS, but not with worse PFS, although it should be noted that the absolute 3-year PFS difference between high- and low-risk groups was nearly 35%. The 3-year OS was 92.3% for low-risk and 68.8% for high-risk patients (χ2 = 10.4, *p* = 0.001, Fig. 1a). The 3-year PFS was 90.0% for low-risk and 55.2% for high-risk patients (χ2 = 2.36, *p* = 0.12).

### Management strategies for Locoregional recurrence

After initial treatment for ACC, the median time to development of LRR was 51 months (IQR, 15–108). Six of these 20 LRR patients were initially treated with surgery alone and 14 patients initially received surgery with PORT. Only eight patients had detailed pathologic information from their primary surgical resection, but all eight had positive margins and PNI, while six had pathologic T4 disease and one patient had > 30% solid histology.

Histopathologic evaluation of LRR after surgical resection of the recurrence was performed for 17 patients and found a median size of 3.0 cm (IQR, 1–3.5), 11 patients with positive margins, 11 with PNI, two with LVSI, and four with solid component. Eleven patients (55%) had high-risk LRR involving the skull base. Treatments for LRR consisted of surgery with PORT in 10 patients (50%), surgery only in eight patients (40%), high-dose radiation only in one patient (5%) and systemic therapy only in one patient (5%). Of the 10 patients receiving PORT, only five received standard-course fractionated external beam radiation; the remaining five received stereotactic radiosurgery (SRS), stereotactic body radiotherapy (SBRT), intra-operative radiation, or external beam radiation using altered fractionation. One patient who received surgery and PORT also received adjuvant chemotherapy. There was no clear association between high-risk LRR and delivery of multimodality therapy although a trend to improved outcome was likely limited by sample size (χ2 = 2.55, *p* = 0.11).

The median follow-up time after LRR diagnosis was 38 months (IQR, 11.5–53.5), with three patients developing re-progression after their treatment for LRR. One patient had a parotid ACC that was re-excised at first recurrence, recurred seven years later, and was re-excised with PORT. Despite durable local control of over 13 years at the parotid, this patient eventually developed lung, spleen, kidney, and soft tissue metastases. One patient had a maxillary ACC initially treated with surgery and PORT, who recurred locally and underwent repeat surgical excision three times in two years before succumbing to recurrent skull base disease. The third patient had a hard palate ACC initially treated with surgery and PORT who developed a pterygopalatine fossa recurrence involving V1 and V2 nerves and was treated with surgery and SBRT, but eventually developed re-progression of local disease and was started on a systemic therapy clinical trial. Lung DM was found concurrently in one patient and was diagnosed eight months after treatment of LRR in a second patient.

Multimodality therapy was not found to be associated with PFS or OS outcomes. We therefore investigated whether older age, size of recurrence, positive margins at time of LRR resection, histopathologic factors of the primary or at the time of recurrence (solid histology, LVSI, PNI), or high-risk LRR were associated with PFS or OS. The only factor associated with PFS was size of recurrence < 3 cm (log rank χ2 = 5.0, *p* = 0.03); Cox proportional hazards ratios could not be calculated due to too few progression events. High-risk LRR was the only variable associated with OS. The 3-year OS was 87.5% for low-risk and 62.3% for high-risk patients (log-rank χ2 = 5.9, *p* = 0.01, Cox HR 10.1, 95%CI 1.1–90.0, *p* = 0.038, Fig. 1b). The 3-year PFS was 100% for low-risk and 64.3% for high-risk patients (χ2 = 2.1, *p* = 0.15).

### Management strategies for metastatic disease

After initial treatment for ACC, the median time to development of DM was 50 months (IQR, 18–77). The initial pathology of the 16 patients with DM was reviewed and two (12.5%) had positive nodes, eight (50.0%) were pT3 or pT4, six (37.5%) had ≥30% solid histological component, and 13 (81.3%) had close or positive margins. Of the 9 patients with core biopsies or surgical specimens of DM available for in-depth pathologic analysis, 2 had solid component subtypes, 0 had PNI, 2 had LVSI, and 1 had a positive margin.

Fourteen patients (87.5%) had recurrent lung metastases, nine of which were classified as IRLM Group III/IV. Other observed sites of metastases included bone, central nervous system, and subcutaneous tissue. In total, 11 patients (68.8%) were considered to have high-risk DM with IRLM Group III/IV or non-lung metastases.

Six patients with small-volume bilateral lung disease were initially managed with observation. Four eventually received treatment with surgery (1), radiation (1), chemotherapy (1), or were enrolled in a clinical trial (1). Of these patients initially deemed safe for initial management by observation, the median OS after recurrence diagnosis was 65.5 months (IQR, 28–100).

Among 10 DM patients receiving upfront treatment, five (50%) received surgery, two (20%) received SBRT, two received systemic therapy only (20%) and one received palliative radiation therapy (10%). The seven patients treated with upfront surgery or non-palliative radiation had a median survival of 45 months (IQR, 28–93) and four were disease-free for the remainder of their follow-up, which ranged from 25 to 51 months.

Upfront treatment with surgery or non-palliative radiation therapy was not found to be associated with PFS or OS. We therefore investigated whether older age, size of dominant DM, positive margins at time of DM resection, histopathologic factors of the primary or at the time of metastasis (solid histology, LVSI, PNI), or high-risk DM were associated with PFS or OS. We found no significant association between pre-treatment or treatment-related factors with PFS. High-risk DM was the only variable significantly associated with OS. The 3-year OS was 100% for low-risk patients compared to 76.2% for high-risk patients (log-rank χ2 = 4.7, *p* = 0.03, Fig. 1c). Cox proportional hazard ratios could not be calculated as no patients with low-risk DM died during the follow-up period (median 37.5 months, IQR 22–64). The 3-year PFS was 80.0% for low-risk and 44.4% for high-risk patients (χ2 = 0.51, *p* = 0.48).

## Discussion

**Fig. 2:**
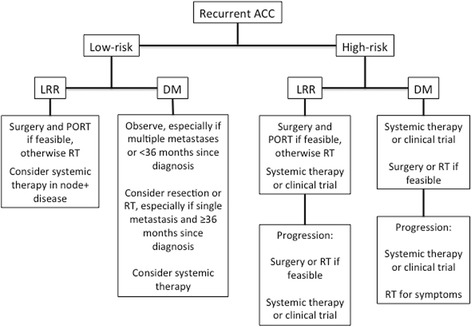
Proposed risk-stratified treatment considerations for recurrent adenoid cystic carcinoma (ACC). LRR = locoregional recurrence. DM = distant metastasis. PORT = postoperative radiation therapy. RT = radiation therapy

Limited data is available to guide the clinical management of locally recurrent or metastatic ACC. We therefore analyzed 36 consecutive cases of recurrent ACC at a single tertiary care institution to describe treatment outcomes and prognostic variables. In LRR, the most common treatments were surgery with or without PORT; high-risk disease at the skull base was often aggressively treated in an attempt to maintain local control. In DM, lung metastases were the most common sites of disease managed with observation, surgery, or SBRT, depending on resectability, bilaterality, and whether the metastases were singular or multiple in number. While no treatment paradigms or histopathologic factors were identified as prognostic for PFS or OS after recurrence, stratifying patients by high- and low-risk classifications found that a high-risk pattern of disease recurrence was associated with significantly decreased survival. Based on our institutional practices, we propose risk-stratified treatment considerations in Fig. 2.

In our study population, the median time to first recurrence (whether LRR or DM) was approximately 50 months from initial treatment. After a recurrence diagnosis, we observed a 3-year OS of 79% overall, with LRR patients having lower Kaplan-Meier estimates of OS (73%) compared to DM patients (85%). Although not significantly different, these findings indicate that the time to LRR is comparable to that of DM with an equally decreased or potentially worse subsequent survival outcome. Our findings are in keeping with observations from large retrospective reviews indicating that local recurrence is a critical issue associated with cause-specific survival. [10, 20]. Yet, whereas many studies have sought to define survival prognostics related to DM [2, 13, 21–23], very little literature exists to describe survival prognostics or management strategies for LRR.

At our institution, LRR was often treated in an aggressive and multimodal fashion (Fig. 2) with 90% of patients receiving resection and 50% of patients receiving PORT. Due to the use of prior full-course radiation, only five patients were able to receive fully fractionated PORT. The other five were re-irradiated using extremely conformal techniques such as SRS, SBRT, or intraoperative radiotherapy. We found that traditional prognostic variables such as age, histology, margin status, LVSI, and PNI were not associated with PFS or OS (size < 3 cm was associated with worse PFS on log-rank testing, but there were too few progression events to determine whether this was a real effect in Cox proportional hazards modeling). However, due to our small sample size and unavailable pathologic information on six specimens, we could not entirely rule out the prognostic value of traditional pathologic characteristics. The only prognostic factor we were able to detect was based on worse survival in LRR from high-risk recurrent disease involving the skull base. While this finding may support an approach of treating skull base LRR early and aggressively, it should be noted that progressive local and metastatic disease continued despite our efforts to maximize local control in these patients. There is an urgent need for more effective combinatorial and systemic therapies to enhance the principal strategy of surgical resection.

Although the patients with DM in this cohort had heterogeneous treatments ranging from observation to metastasectomies to chemotherapy, the observed outcomes remained consistent with those reported in literature. The majority of patients with DM (80%) had lung metastases [1, 2, 8, 12]. Many with low-volume lung disease were initially managed with observation and these carefully selected patients did not have significantly poorer outcomes compared to upfront surgery or non-palliative radiation [15, 24]. Of patients who received metastasectomies or non-palliative radiation, 4 of 7 (57%) enjoyed disease-free intervals of 2–4 years.

We confirmed that patients with extrapulmonary [12, 21] or IRLM Group III/IV lung metastases [18] constitute a high-risk group with significantly worse OS outcomes. The inverse was also true, as we found that the low-risk group was associated with a good prognosis with no deaths observed in the follow-up period.

When managing lung metastases, our institution considers resectability, number of metastases, bilaterality, and pace of disease (Fig. 2). In patients with Group III or IV lung metastases (high-risk DM), we favor starting with systemic therapy with limited resection or radiation if safe and feasible, or for palliation. In patients with Group I low-risk DM (resectable, DFI ≥36 months, and single metastasis), we consider this to represent oligometastatic disease and favor early metastasis-directed therapy with resection or radiation. In Group II low-risk DM (resectable, DFI < 36 months or multiple metastases), we favor observation with ascertainment of disease stability before deciding upon a course of further observation, resection/radiation, or initiation of systemic therapy. This is supported by our findings that initial observation for low-volume bilateral lung disease did not have significantly poorer outcomes compared to upfront treatment.

Overall, in the two cohorts of patients experiencing LRR and DM, an initial recurrence with high-risk disease was highly associated with OS (*p* = 0.001). Therefore, for patients with skull base recurrences, extrapulmonary metastases, or multiple lung metastases that develop within 36 months of initial treatment, better prognostic markers and effective treatments are urgently needed.

Our study is limited by small sample size, heterogeneous treatment paradigms, and selection bias as a tertiary care institution. To maximize completeness of the analysis, we restricted our population to a cohort of patients whose recurrences were treated and followed at our institution, but this may have introduced biases related to the patient population and/or our treatment policies. Some patients did not have full pathologic characteristics available at time of recurrence. Slides from the initial surgical specimen may not always be available, as recurrences at our institution are often diagnosed by fine needle aspiration (thereby limiting pathologic analysis of PNI and LVSI) and/or are treated with non-surgical approaches (thereby limiting available tissue for pathologic analysis). Where possible, we maximized the pathologic information available to us, but further studies incorporating full pathologic review and confirmation of these findings would be useful.

## Conclusion

Recurrences, both local and distant, can be life-limiting in patients with ACC. At our institution, LRR is commonly managed with surgical excision and PORT. Metastases are managed heterogeneously with observation, metastasectomy, radiation, or systemic therapy depending on number, bilaterality, and resectability. We developed a set of criteria designating high-risk disease (skull base recurrence, extrapulmonary metastases, and IRLM Group III/IV lung metastases), which were significantly prognostic of OS; enhanced treatments are needed for these patients. Additional studies are needed to validate these findings and identify better prognostic markers and therapeutics.

## Abbreviations

ACC: Adenoid Cystic Carcinoma
DFI: Disease-free interval
DM: Distant metastases
IQR: Interquartile range
IRLM: International Registry of Lung Metastases
LRR: Locoregional recurrence
LVSI: Lymphovascular space invasion
OS: Overall survival
PFS: Progression-free survival
PNI: Perineural invasion
PORT: Postoperative radiation therapy
SBRT: Stereotactic body radiotherapy
SRS: Stereotactic radiosurgery
